# DNA Recombination and Repair—A New Twist to RecA Function

**DOI:** 10.1371/journal.pbio.0030070

**Published:** 2005-02-08

Molecular motors harness the energy of ATP (or GTP, a related energy currency) and transform it into mechanical force. Well-known examples of motors include myosin and dynein, proteins that use ATP to ferry intracellular cargo along fibers made of actin or tubulin proteins. The ATP-dependent assembly of actin or tubulin fibers itself can work as a motor: for instance, the march of white blood cells toward pathogens is powered by the growth of actin filaments pushing against the cells' membranes. In all cases, coherent motion implies a coordinated and polarized use of energy.

Now, Julia Cox, Oleg Tsodikov, and Michael Cox present evidence indicating that filaments of the bacterial RecA protein, long known for their role in homologous recombination and DNA repair, have properties reminiscent of a molecular motor as well. RecA filaments consist of DNA helices lined with RecA protein. RecA filaments invade a region of double-stranded DNA with similar nucleotide sequence, displacing one strand to pair with the other. Strand invasion can lead to a re-assortment—known as recombination—of DNA regions on either side of the shared sequence. It can also initiate the repair of DNA lesions during replication—the process by which a DNA molecule is copied to make two.

RecA is also an ATPase, an enzyme capable of hydrolyzing (breaking down) ATP, when bound to DNA. RecA uses ATP to carry out strand exchange over long sequences and impose direction to the exchange, to bypass short sequence heterogeneities, and to stall replication so DNA lesions can be mended. But how RecA molecules within a filament coordinate and organize their activities to carry out these functions has remained obscure.

Cox et al. addressed this problem in the test tube, by examining RecA filaments grown from mixing RecA protein with DNA. Previous experiments have shown that filament assembly spreads rapidly in the 5′-to-3′ direction once the first RecA molecule is loaded onto DNA. At the same time, ATP hydrolysis causes the release of RecA from DNA, but the exact rate of RecA dissociation is not known. Experiments suggest, however, that RecA molecules only dissociate from DNA when they are at the fiber's 5′ end, while ATP hydrolysis occurs all along its length.[Fig pbio-0030070-g001]

**Figure pbio-0030070-g001:**
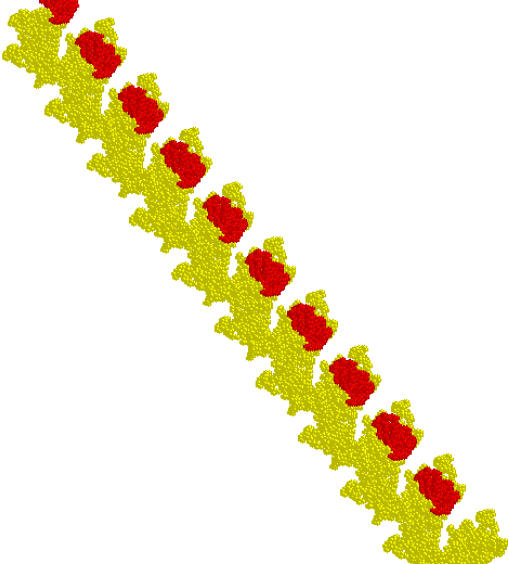
A RecA filament with every sixth molecule in red

Under their experimental conditions, the authors found that a RecA molecule hydrolyzed 20 ATPs per minute—or one ATP every three seconds. If ATP hydrolysis occurred randomly in the fiber, one would expect a RecA molecule to dissociate about once every 1.5 seconds, or 40 RecA molecules to dissociate per minute. But this is not what the authors found—instead they estimated the dissociation to be at a rate of 120 RecA molecules per minute. Hence, six RecA molecules dissociate from a fiber in the time—three seconds—it takes for an individual RecA molecule to burn one ATP. This implies a filament organization in which every RecA molecule hydrolyzes ATP in synchrony with the sixth RecA molecule to its left and the sixth RecA molecule to its right.

The authors note that there are approximately six RecA molecules per helical turn in a RecA filament. They propose that the RecA molecules hydrolyzing ATP at any given moment are aligned in a “stripe” that runs along the side of the filament. This stripe of ATP hydrolysis moves around the fiber in a repeating pattern of six steps. At the 5′ end of the fiber, ATP hydrolysis leads to RecA release. But in the middle of the fiber, it could work as a rotary motor, with the power to wind or unwind DNA and drive strand invasion through difficult passages of damaged DNA.

